# Periconceptional folic acid supplementation is a risk factor for childhood asthma: a case-control study

**DOI:** 10.1186/s12884-022-04567-5

**Published:** 2022-03-18

**Authors:** Shuyuan Chu, Jun Zhang

**Affiliations:** 1 Laboratory of Respiratory Disease, Affiliated Hospital of Guilin Medical University, Guilin, 541001 China; 2 MOE-Shanghai Key Laboratory of Children’s Environmental Health, Xinhua Hospital, School of Medicine, Shanghai Jiao Tong University, Shanghai, 200092 China

## Abstract

**Background:**

Several studies found an association between periconceptional folic acid supplementation and the risk of childhood asthma. But the epidemiologic evidence is still inconsistent and the underlying biological mechanisms remain unclear.

**Methods:**

We conducted a hospital-based case-control study on childhood asthma with 548 cases and 816 normal controls in Shanghai, China. Mothers of the asthma children were asked about folic acid supplementation before and during pregnancy. Unconditional logistic regression models were employed to control for potential confounders.

**Results:**

Periconceptional folic acid supplementation was associated with an increased risk of childhood asthma after adjusting for potential confounders (adjusted OR = 1.28 [95% CI 1.14–1.43]). Moreover, the adjusted OR varied by the timing of starting folic acid supplementation: before gestation: 1.31 [95% CI 1.01–1.70]; in the 1st month of gestation: 1.09 [95% CI 0.96–1.23]; and after the 1st month of gestation: 1.90 [95% CI 1.56–2.30]. We further found that the adjusted OR was the highest when periconceptional folic acid supplementation lasted more than 6 months (< 4 months: 1.21 [95% CI 1.07–1.37]; 4–6 months: 1.06 [95% CI 0.88–1.27]; > 6 months: 1.75 [95% CI 1.35–2.27]).

**Conclusions:**

Periconceptional folic acid supplementation was associated with an increased risk of childhood asthma in offspring. Further research on this issue is warranted.

## Background

Periconceptional folic acid supplementation has been recommended by the World Health Organization to prevent neural tube defects [1]. Interestingly, folic acid provides methyl that could induce epigenetic changes by altering the status of methylation-sensitive, and then enhance the expression of T-helper type 2 (Th2) cytokines during fetal development. The latter may change the inflammatory response and the risk of allergic airways disease in offspring [2, 3]. Thus, it is at least theoretically possible that periconceptional folic acid supplementation may increase the risk of asthma in offspring [4]. However, the conclusions in previous studies were inconsistent. For example, several studies [5–8] reported an association between prenatally exposed to folic acid and an increased risk of childhood asthma while others [9–11] failed to confirm. Therefore, we tested this hypothesis in a hospital-based case-control study.

## Methods

A detailed description of the hospital-based case-control study protocol has been provided elsewhere [12]. Briefly, children aged 4 to 12 years were recruited in the Xinhua Hospital, Shanghai, China, from June 2015 to January 2016. Childhood asthma was diagnosed by pediatrician according to the Global Initiative for Asthma guidelines [13]. The controls were non-asthma children from outpatient clinics. The potential asthmatic children in controls were excluded by using a wheezing module from the International Study of Asthma and Allergies in Childhood [14]. The study was approved by the Committee of Research Ethics at the Xinhua Hospital and conformed to the Declaration of Helsinki. All parents signed an informed consent.

Information was collected from parents by a face-to-face interview, which included parental demographics, environmental exposure, periconceptional folic acid supplementation, and the start time and duration of folic acid supplementation. The folic acid supplementation was supplementing folic acid 400–800 micrograms every day by taking folic acid tablets or vitamin with it. The start time of folic acid supplementation was between 6 months before gestation and 6 months after gestation. The longest duration was 12 months. We explored the associations between periconceptional and gestational exposure to folic acid and risk of childhood asthma. We further examined the effect of start time (before gestation, during the 1st month, or > 1st month of gestation) and duration (< 4, 4–6, or > 6 months) of maternal folic acid supplementary on the childhood asthma in the analysis.

We adjusted covariates in the models as following: maternal education levels (≤9, 10–12, 13–16, or ≥ 17 years), paternal education levels (≤9, 10–12, 13–16, or ≥ 17 years), family history of allergic diseases in any of his family members (no/yes), child age, gender, birthweight, gestational age, delivered by caesarean section, newborn resuscitation (no/yes), and feeding in the first 6 months (breast feeding, or mixed or exclusive formula feeding). Those covariates were selected as described before [12]. Missing data were assigned to a separate category in models. Odds ratios (OR) and 95% confidence intervals (CI) were calculated using unconditional logistic regression models with LOGISTIC procedure in SAS 9.4 (SAS Institute Inc., Cary, NC, USA).

## Results

**Fig. 1:**
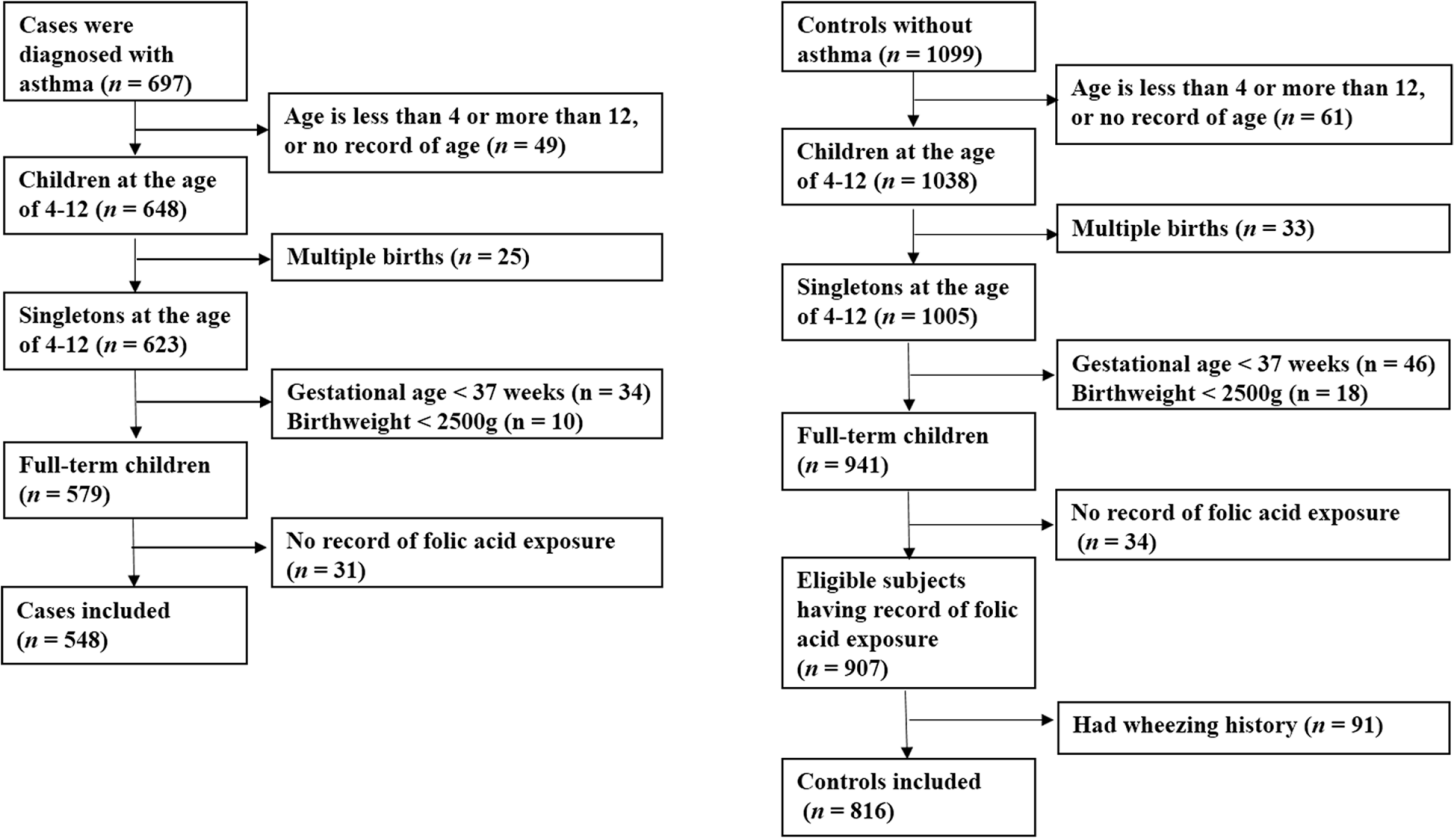
Population flow chart of the case-control study

Figure 1 illustrates the subject selection process. We excluded cases and controls if they were not between 4 to 12 years of age or had missing information on age, twins, preterm births or low birth weight, or had no information on maternal folic acid supplementation. Controls with history of wheezing were also excluded, leaving a total of 548 cases and 816 controls for final analyses.

The case group had a significantly higher proportion of periconceptional folic acid supplementation before and during pregnancy compared with the control group (*p* < 0.01) (Table 1). The cases were more likely to be boys, younger, delivered by caesarean section, with a higher proportion of family history of allergic diseases and higher parental education.

**Table 1:** Demographic, perinatal and child characteristics in the case-control study

Characteristics	Case (***n*** = 548)		Control (***n*** = 816)	
	No.	%	No.	%
Gender (boys)**	333	60.8	420	51.5
Age**				
4	197	35.9	208	25.5
5–6	205	37.4	274	33.6
7–8	88	16.1	173	21.2
9–10	44	8.0	129	15.8
11–12	14	2.6	32	3.9
Family history of allergic diseases**	280	51.1	165	20.2
folic acid exposure**	436	79.6	553	67.8
**Birth weight**				
2500–2999	77	14.1	113	13.8
3000–3499	264	48.2	401	49.1
3500–3999	160	29.2	240	29.4
≥ 4000	47	8.6	62	7.6
**Newborn resuscitation**	11	2.0	19	2.3
Caesarean section*	342	62.4	465	57.0
**Feeding in the first 6 months**				
Breast feeding	293	53.5	442	54.2
Mixed or exclusive formula feeding	253	46.2	374	45.8
Passive smoking**	188	34.3	382	46.8
Maternal educational level (yrs)**				
≤ 9	37	6.8	149	18.3
10–12	63	11.5	143	17.5
13–16	351	64.1	405	49.6
≥ 17	44	8.0	40	4.9
Paternal education level (yrs)**				
≤ 9	33	6.0	134	16.4
10–12	59	10.8	146	17.9
13–16	340	62.0	400	49.0
≥ 17	61	11.1	59	7.2

*: *P* < 0.05

**: *P* < 0.01

Table 2 further illustrates that periconceptional folic acid supplementation was associated with an increased risk of childhood asthma after adjusting for potential confounders (adjusted OR = 1.28 [95% CI 1.14–1.43]). Moreover, the adjusted OR was the highest when the folic acid supplementation was started after the first month of gestation (before gestation: adjusted OR = 1.31 [95% CI 1.01–1.70]; 1st month of gestation: adjusted OR = 1.09 [95% CI 0.96–1.23]; >1st month of gestation: adjusted OR = 1.90 [95% CI 1.56–2.30]). We further found that the adjusted OR was the highest when the supplementation lasted more than 6 months (< 4 months: adjusted OR = 1.21 [95% CI 1.07–1.37]; 4–6 months: adjusted OR = 1.06 [95% CI 0.88–1.27]; > 6 months: adjusted OR = 1.75 [95% CI 1.35–2.27]).

**Table 2:** Adjusted and unadjusted relative risks of asthma in children periconceptionally and gestationally exposed to folic acid

Exposure	case	control	Unadjusted OR	95% CI	Adjusted OR *	95% CI
**Folic acid exposure**						
No	112	263	Ref.	\	Ref.	\
Yes	436	553	1.77	1.60–1.96	1.28	1.14–1.43
**Starting time**						
No exposure	112	263	Ref.	\	Ref.	\
Before gestation	278	372	1.64	1.47–1.82	1.31	1.01–1.70
1st month of gestation	62	85	1.57	1.33–1.85	1.09	0.96–1.23
> 1st month of gestation	26	32	2.20	1.74–2.79	1.90	1.56–2.30
**Duration**						
No exposure	112	263	Ref.	\	Ref.	\
< 4 months	28	32	1.81	1.43–2.29	1.21	1.07–1.37
4–6 months	269	400	1.47	1.32–1.63	1.06	0.88–1.27
> 6 month	62	60	2.40	2.03–2.85	1.75	1.35–2.27

*: adjusted for paternal education level, family history of allergic diseases, age, gender, birthweight, gestational age, delivered by caesarean section, newborn resuscitation, and feeding in the first 6 months

## Discussions

Our study shows that folic acid supplementation during pregnancy was associated with an increased risk of childhood asthma in offspring. The risk was particularly high when the supplementation was started after the first month of gestation and lasted for more than 6 months.

Our findings were consistent with some previous studies which suggested that periconceptional folic acid supplementation might increase the risk of childhood asthma [5–8]. For example, Veeranki and colleagues found that children born to women with periconceptional folic acid supplementation had increased relative odds of asthma (adjusted OR = 1.2) [7]. However, this association was not found in some cohort studies [9–11]. Folic acid acts as a methyl donor which is an important source of methyl groups for DNA methylation [15]. Some studies suggest that exposure to methyl donors in utero could affect T-helper (Th) cell development and skew it into Th2, resulting in an increased susceptibility to asthma and allergy diseases [3, 16].

We also found that starting supplementation after the first month of pregnancy was associated with a highest risk of childhood asthma. Moreover, the adjusted OR is highest when periconceptional folic acid supplementation lasted more than 6 months. Our findings are similar to the previous finding in that starting supplementation in late pregnancy was associated with an increased risk of childhood asthma at 3.5 years (relative risk (RR) = 1.26, 95% CI = 1.08, 1.43) [5]. While the biological mechanisms are unknown, we speculate that these associations may be related to immune system development. T cells begin to develop at the 12 weeks of gestation [17] while IgE receptor activity rises greatly in the fetus during 16–20 weeks of gestation [18]. IgE production is stimulated by Th12. Our findings suggest that folate intake in very early gestation and lasting less 6 months might be safer.

Our study has some limitations. First, it drew on a hospital-based sample. It may be suspected that wealthier, better-educated families might be more likely to bring their children with asthma symptoms to hospital. These families may also be more likely to supplement folic acid during pregnancy [19, 20]. Thus, even though we have adjusted for maternal and paternal education, it is still possible that residual confounding may have affected our results. However, this speculation is not consistent with our finding that supplementation starting after 1st month of pregnancy is associated with the highest risk because better educated women tend to take folic acid earlier rather than later. Second, information on periconceptional folic acid supplementation was self-reported. In our study, the prevalence of folic acid supplementation was 73% (989/1364), which was similar to a previous survey in Shanghai (71%) [21]. In addition, 48% (650/1364) of our subjects took folic acid supplementation for less than 3 months. That is similar with previous reports in Beijing, in which 53.6% of pregnant women used folic acid supplementation during the first 3 months after pregnancy [22]. Thus, the bias of information on folic acid supplementation may not be a serious issue in our study. Third, the dietary intake of folate wasn’t assessed in this study. Since all subjects were from the same district, the mothers had the same dietary habit. Based on the survey in east south of China, the dietary folate intake among women of childbearing age was 205–225 micrograms every day [23]. Thus, the folate intake from diet could be supposed to be similar between the two groups. And folic acid food fortification has not been introduced in China. Therefore, by matching the control group, the effect of folic acid supplementation should be from supplementing folic acid tablets or vitamin with it.

## Conclusions

Periconceptional folic acid supplementation was associated with an increased risk of childhood asthma in offspring in China. Supplementation starting after the first month of gestation, or lasting more than 6 months was associated with the highest risk of childhood asthma in offspring. Our findings may have important clinical and public health implications in recommending that folate intake before or during the first month of gestation and for less 6 months might be safer. More research on this important issue is warranted.

## Data Availability

The dataset generated and/or analyzed during the current study are not publicly available but are available from the corresponding author on reasonable request.

## References

[1] http://www.who.int/elena/titles/folate_periconceptional/en/. Accessed on July 12, 2018.

[2] Waterland RA, Michels KB (2007). Epigenetic epidemiology of the developmental origins hypothesis. Annu Rev Nutr.

[3] Hollingsworth JW, Maruoka S, Boon K, Garantziotis S, Li Z, Tomfohr J (2008). In utero supplementation with methyl donors enhances allergic airway disease in mice. J Clin Invest.

[4] Crider KS, Cordero AM, Qi YP, Mulinare J, Dowling NF, Berry RJ (2013). Prenatal folic acid and risk of asthma in children: a systematic review and meta-analysis. Am J Clin Nutr.

[5] Whitrow MJ, Moore VM, Rumbold AR, Davies MJ (2009). Effect of supplemental folic acid in pregnancy on childhood asthma: a prospective birth cohort study. Am J Epidemiol.

[6] Zetstra-van der Woude PA, De Walle HE, Hoek A, Bos HJ, Boezen HM, Koppelman GH (2014). Maternal high-dose folic acid during pregnancy and asthma medication in the offspring. Pharmacoepidemiol Drug Saf.

[7] Veeranki SP, Gebretsadik T, Mitchel EF, Tylavsky FA, Hartert TV, Cooper WO (2015). Maternal folic acid supplementation during pregnancy and early childhood asthma. Epidemiology.

[8] Yang L, Jiang L, Bi M, Jia X, Wang Y, He C (2015). High dose of maternal folic acid supplementation is associated to infant asthma. Food Chem Toxicol.

[9] Magdelijns FJ, Mommers M, Penders J, Smits L, Thijs C (2011). Folic acid use in pregnancy and the development of atopy, asthma, and lung function in childhood. Pediatrics.

[10] Martinussen MP, Risnes KR, Jacobsen GW, Bracken MB (2012). Folic acid supplementation in early pregnancy and asthma in children aged 6 years. Am J Obstet Gynecol.

[11] Trivedi MK, Sharma S, Rifas-Shiman SL, Camargo CA, Weiss ST, Oken E (2018). Folic acid in pregnancy and childhood asthma: a US cohort. Clin Pediatr (Phila).

[12] Chu S, Chen Q, Chen Y, Bao Y, Wu M, Zhang J (2017). Cesarean section without medical indication and risk of childhood asthma, and attenuation by breastfeeding. PLoS One.

[13] http://www.ginasthma.org/. Accessed on May 10, 2015.

[14] Asher MI, Keil U, Anderson HR, Beasley R, Crane J, Martinez F (1995). International study of asthma and allergies in childhood (ISAAC): rationale and methods. Eur Respir J.

[15] Niculescu MD, Zeisel SH (2002). Diet, methyl donors and DNA methylation: interactions between dietary folate, methionine and choline. J Nutr.

[16] Larche M (2007). Regulatory T cells in allergy and asthma. Chest.

[17] Rechavi E, Lev A, Lee YN, Simon AJ, Yinon Y, Lipitz S (2015). Timely and spatially regulated maturation of B and T cell repertoire during human fetal development. Sci Transl Med.

[18] Thornton CA, Holloway JA, Popplewell EJ (2003). Fetal exposure to intact immunoglobulin E occurs via the gastrointestinal tract. Clin Exp Allergy.

[19] Lai J, Yin S, Ma G, Piao J, Yong X (2007). The nutrition and health survey of pregnant women in China. Acta Nutrimenta Sin.

[20] Liu FL, Zhang YM, Parés GV, Reidy KC, Zhao WZ, Zhao A (2015). Nutrient intakes of pregnant women and their associated factors in eight cities of China: a cross-sectional study. Chin Med J.

[21] Mi M. Analysis of intakes of diet and nutrients supplements of pregnant women in Shanghai. *J Hyg Res.* 2008; 37(4):460–462. (宓铭. 上海市孕妇营养素补充剂摄入情况分析. 卫生研究)

[22] Hou S, Yang J. Folic acid use and its interfering factors in 20581 pregnant women in Tongzhou District of Beijing. Chinese J Reprod Health 2015;26(6):536–539. :10.3969/j.issn.1671-878X.2015.06.011 (侯杉杉, 徐洁. 北京市通州区20581例产妇孕期服用叶酸情况及影响因素. 中国生育健康杂志. )

[23] Zhao Y, Hao L, Zhang L (2009). Plasma folate status and dietary folate intake among Chinese women of childbearing age. Matern Child Nutr.

